# Under-Age Children Returning From Jihadist Group Operation Areas: How Can We Make a Diagnosis and Construct a Narrative With a Fragmentary Anamnesis?

**DOI:** 10.3389/fpsyt.2020.00149

**Published:** 2020-03-26

**Authors:** Anaelle Klein, Alessandra Mapelli, Maurween Veyret-Morau, Julie Levy-Bencheton, François Giraud, Mercedes di Chiara, Marta Fumagalli, Hélène Lida-Pulik, Ana Moscoso, Jérôme Payen de la Garanderie, Stephanie Palazzi, Jean-Marc Baleyte, Mario Speranza, Dalila Rezzoug, Thierry Baubet

**Affiliations:** ^1^Child and Adolescent Psychiatry Department, AP-HP, Avicenne Hospital, EA 4403 Paris 13 University, Bobigny, France; ^2^University Paris-Saclay, UVSQ, INSERM, CESP, Team « PsyDev », Villejuif, France; ^3^Psychiatry and Addictology Department, APHP, Bichat Beaujon (Pr M. Lejoyeux), University of Paris, Paris, France; ^4^Child and Adolescent Psychiatry Department, Intermunicipal Hospital Center of Creteil, Créteil, France; ^5^Child and Adolescent Psychiatry Department, Versailles General Hospital, Versailles, France; ^6^EA4047 Versailles-Paris Saclay University, Versailles, France

**Keywords:** child returnees, stress disorders, traumatic, war exposure, terrorism

## Abstract

**Introduction:**

Since 2011, the French government estimates that about 500 French children have been born in or taken by their parents to areas where terrorist operations prevail. Since May 2017, 75 children who returned to France have benefited from a dedicated health care system.

**Method:**

This article is the result of clinical interviews conducted with 53 patients evaluated and taken care of at Avicenne Hospital in Bobigny. To our knowledge, no studies have been published on this subject.

**Results:**

A total of 32 evaluations have been completed, all of which indicated the need for care for these children. Of these children, 64% are under 5 years old, and 59% were born in France. Their clinical profiles are heterogeneous and fluctuate with time.

**Discussion:**

The multiple adverse events experienced by these children and the uniqueness of children born to families suspected by authorities of having participated in activities related to terrorism make this situation unprecedented. How can we make a diagnosis of PTSD without the help of a precise anamnesis? How can we help these children form a structuring narrative that avoids the pitfalls inherent to generalized fascination?

## Introduction

The French government estimates that about 500 under-age children were born in the Iraqi-Syrian war zone or taken there by their parents (1). France is the European country with the highest number of under-age children who were raised under the “Islamic state” (2). The return of emigrated families to their countries of origin is a major challenge for governments and actors in the field.

Faced with this exceptional and unprecedented situation, the French Government implemented a dedicated support system for the return of these children (1, 3). It has several different components: security, child protection, health, and education. The health component is independent of the others, in compliance with the rules of medical ethics.

Since May 2017, 75 children returning from Iraq, Syria, or other terrorist war zones (Afghanistan, Mali, Yemen, etc.) have benefited from this system. The vast majority of these children (74%) were taken care of by the Avicenne medical team. The purpose of this article is to report on the particularities of this on-site care. First, we will describe the dedicated support system implemented by the French government for the return of children from the jihadist group operation areas; secondly, we will present some data concerning the clinical profiles and questions raised by the first wave of children assessed by the Avicenne psychiatric team around the diagnosis, the construction of a story, and the interinstitutional link.

To our knowledge, no studies have previously been published on this subject.

## Materials and Methods

The present article is based on the expertise emanating from our clinical encounters. It is the result of the care of the 53 patients assessed and managed at the Avicenne site between March 2017 (the date of the first patients being taken care of, just before the installation of the dedicated support system) and June 2019. We present data and reflections that have issued from our clinical assessment. Data were collected by psychologists and child psychiatrists as part of their clinical care, and reflections arise from discussions in our team meetings. We have not yet been able to carry out an epidemiological study because of the specific context, the low sample number, and the age of the children. Therefore, the agreement of an ethics committee is not required. Due to the many pitfalls involved in completing standardized questionnaires for this specific clinic, we conducted a systematized clinical assessment of the child with detailed note-taking, and the diagnosis was made by the consensus of the clinical group.

## Results

### Description of the Dedicated Support System

France is the first country to have implemented a systematized care scheme for the assessment and care of minors returning from jihadist group operation areas. Three university centers have been designated in the Paris region, in Bobigny, Créteil, and Versailles, each including a child and adolescent psychiatry department and a pediatrics department.

Two Prime Ministers have drafted instructions specifying the measures to be implemented by every state service for the return or arrival of these children.

The majority of the children return with a parent, most often their mother. In many situations, fathers are dead or presumed dead. In some cases (parents deceased or incarcerated in the area), the children are brought to France by a humanitarian association. In both cases, when entry into France is scheduled, upon arrival at the airport, they are separated from their parents (when accompanied by them). The parent(s) is (are) placed in police custody and pre-trial detention upon their arrival.

The Paris Public Prosecutor’s Office coordinates the system and refers the matter to a juvenile judge. The latter entrusts the children to the Child Protective Services (CPS) and requests a socio-educational investigation of the child, of the parents, and of the extended family (grandparents, uncles, aunts, etc.) to assess the possibility of the child’s return to his or her family, as well as their capacity to accommodate the children.

The judge can place the child in a children’s shelter or, more often, with a foster family. In conjunction with the social services, the judge decides on the subsequent general care.

For children born in France or in an internationally recognized country, the parental authority of a parent is maintained. Mediatized visits to prisons, in the presence of children’s professionals of the educational services, are organized at a variable frequency in order to maintain a strong parent–child relationship. The judge may also authorize a stay with the extended family.

On the other hand, for children born in areas where terrorist groups prevail, their filiation is not always established upon their return to France, as they do not have an internationally recognized civil status. Genetic explorations of the child and those hypothesized to be most likely to be their parents can be asked for by the juvenile judge to establish filiation.

Before the child’s arrival in France, parents sign a consent form for a medical checkup and may provide information on the child’s conditions and lifestyle (breastfeeding, allergies, sleep, type of relationship with parent(s)/siblings, etc.), but this information is not always completed or transmitted (in Avicenne, this assessment had been completed for only three out of 32 patients (9%)]. A medical examination is conducted at the airport to avoid emergencies, and a day of pediatric hospitalization is organized during the first few weeks following their arrival. A child psychiatry assessment is carried out within the framework of weekly consultations, over a period of 3 months, by a psychologist or child psychiatrist. Children are then placed into medium- or long-term follow-up if necessary and referred to other health professionals if needed (speech therapist, psychomotor skills, therapeutic group, etc.)

Children who have grown up in war zones may have been confronted with multiple adverse and traumatic events in their early childhood: exposure to traumatic images, bombardment, violent death of one or more family members, incarceration before transfer to France, uprooting, and severing ties. In most cases, upon assessment, we have very little information about these children’s histories. To date, for the health care professionals, meeting with the parents in prison is not implemented, as it still raises clinical, ethical, and institutional questions. The anamnestic elements are reported to us through our partnerships, mainly *via* the educational services that meet with parents in prison, organize mediatized visits, and make the connection between parents and the health service.

In addition to the symptomatic assessment of the child, assessment of the child’s relationship with the foster family is essential. At each consultation, we meet the foster family with the child to collect the child’s symptomatology at home, to support the creation of a bond, and to identify any possible difficulties.

### Description of the Avicenne Sample

Between March 2017 and June 2019, 56 children were referred to the Avicenne Hospital; 53 had at least one consultation in the department, and seven were redirected to the other centers. To this day, Créteil and Versailles have taken care of six and 20 children, respectively.

Among the 53 children received at the Avicenne hospital, 32 assessments were completed and 21 are being evaluated, all of which indicated the need for care for these children (see **Table 1**).

**Table 1:** Number of children that benefited from the dedicated health care system (May 2017 – June 2019).

Variable	Number of children
Supported by the health care system	75
Within Avicenne Hospital :	
Adressed :	56
Attended consultation :	53
Being evaluated	21
Redirected to the other centers:	7
Completed the evaluation:	32

The 56 children referred to Avicenne hospital were between 2 months and 17 years old when they arrived (4). Of these, 37% were under 2 years old, 27% between 3 and 5 years old, and 29% between 6 and 12 years old. Adolescents (13–17 years old) form a minority group (7%). In terms of place of birth, 59% were born in France, 30% in Iraq-Syria, and 11% in another country (see **Table 2**).

**Table 2:** Demographic characteristics of the 56 children referred to the psychopathology department of Avicenne Hospital between March 2017 and June 2019.

	Number	%
**Age of children on arrival in France**		
0-2 years old	21	37%
3-5 years old	15	27%
6-12 years old	16	29%
13-17 years old	4	7%
**Child’s place of birth**		
France	33	59%
Iraqi-Syrian zone	17	30%
Other internationally recognized country	6	11%

In children, clinical profiles are heterogeneous and highly variable. The disorders observed are also encountered in other situations where children have experienced trauma and separation from the main caregiver.

During the first assessment interview (see **Table 3**), we observed sleep or eating disorders and acute stress states. These symptoms may be secondary to separation from the parent(s), relocation and migration to France, adaptation to the foster family, etc. Separation in placement situations is in itself a potentially traumatic event, especially if it has not been adequately prepared for, which is the case in the majority of our cohort. Of course, not everyone will develop the symptoms of PTSD.

**Table 3:** List of symptoms observed among the 32 child returnees by the Avicenne team.

**Main symptoms observed at initial assessment**	Sleep disorders
	Separation anxiety disorders, acute stress states
	Eating disorders
	Global developmental delay
	Language delays
	No symptoms
**Main symptoms/diagnoses observed at 3 months**	Attachment disorders
	Depressive disorders
	Anxiety disorders
	Adjustment disorders
	Posttraumatic stress disorder (PTSD) or complex PTSD
	No symptoms

We often receive children very soon after separation from their parents. This therefore induces a specific symptomatology, which is added on. The diagnosis of acute stress disorder is then made. These acute stress states may subsequently improve or persist. The clinical profile of these children varies widely: some children have no symptoms and seem perfectly adapted. Other children have global developmental or language delays. Others, on the contrary, show a good level of development, with skills appropriate for their age group.

At the end of the assessment (see **Table 3**), attachment disorders, depressive episodes, anxiety disorders, and adjustment disorders are among the most frequent disorders identified in these young patients. The diagnosis of post-traumatic stress disorder (PTSD) or complex PTSD is often difficult to confirm because of a lack of anamnesis. Some patients initially exhibit very few symptoms over the 3-month period, but after many months of follow-up, following an event such as a visit to the prison or a change of living quarters, symptoms are triggered in a delayed manner that were initially absent. This observed fluctuation in the disorders exhibited is classic in the trauma clinic (5). The persistence of separation from parents continues, of course, to affect the clinical expression of disorders, even in the long term, but so do the many disruptions that affect the child: visits to prison, meetings with sometimes unknown family, the start of schooling, etc.

Acute symptomatology is therefore not necessarily predictive of a chronic disorder. The absence of an acute disorder does not guarantee a favorable course: symptoms of a likely delayed “PTSD” (5, 6) may appear. Evaluation over a long period of time is necessary to sharpen our eyes and make a stable and reliable diagnosis.

## Discussion

The unprecedented context of children returning from a terrorist war zone gives rise to several questions at the clinical level but also at the level of the general system that surrounds the child. As a preamble, it should be recalled that the diagnosis of PTSD obviously does not summarize all the clinical symptomatology of the child secondary to exposure to one or more traumatogenic events and caregiving ruptures. This diagnosis should be used with caution in children under 12 months of age (7). Nevertheless, we chose the PTSD diagnosis as an example to illustrate the diagnostic difficulties encountered in this situation. Searching for the presence of this diagnosis has been defined as the first objective of the child psychiatric assessment in the system (1).

This support system for minors returning from an area where terrorist groups prevail is unique for several reasons: first, we have very little information on the child’s anamnesis of their first years of life. Unlike most children we assess in a traditional child psychiatric setting, we do not have access to the history of parent/child interactions, nor to parents’ stories. When the parents are in prison, the known elements are reported to us by the educational services, who meet the parents or have access to documents in the judge’s file. Parents’ comments are therefore not collected directly by our team. Professionals who meet with parents note that parents frequently seem to minimize their children’s traumatic exposures. In the majority of cases, we therefore know little about the child’s previous condition before the separation from his or her mother. Cultural factors shape the expression of psychiatric symptoms in children (8). These children were raised in French families, in the context of Islamic state, and little is known about their environment. In many cases, not only do we not have access to the parents but also to the extended family, carriers of the child’s cultural cradle. We therefore work with the descriptions of the educators who met them.

However, in order to diagnose PTSD, the first criterion is to know the history of exposure to traumatogenic events. Indeed, according to DSM-5, the first criterion for children under 6 years of age is “to have faced death or a threat of death, serious injury, or sexual violence” (9). This first exposure criterion is also used in the DC classification: 0–5 (7), according to whether the child experienced the traumatic event, heard or saw the event occur to others, or learned that an event of this type had occurred to a person in the child’s immediate environment. Following exposure to this event, the onset or reactivation of a typical symptomatology of PTSD in children is observed. However, for most of the patients we met, we do not have access to the anamnesis elements that are so necessary for diagnosis. We very rarely know if the symptoms observed existed before their arrival in France. Also, we do not know the reaction of the parents or adults present when the child was exposed to potential traumatic events. The diagnosis of adaptation disorder is therefore often retained by default. It can be very difficult to distinguish PTSD from persistent complex bereavement disorder, especially in young children. Sometimes a child may develop both posttraumatic stress disorder (PTSD) and persistent complex bereavement disorder. In many situations, the father is “missing” or “presumed dead” because the body has not been formally identified. In this case, it is very difficult to make a diagnosis.

The use of standardized questionnaires to assess trauma is problematic at different levels. Almost all scales or diagnostic structured interviews that assess children’s PTSD (10–14) require access to this history. Should it be considered that having been raised in a so-called “war zone” is sufficient exposure to enlist the diagnosis (11)? For our patients, it is difficult to generalize exposure, which must always be assessed individually, according to the exact living space in the area. The vast majority of diagnostic scales or structured interviews are for children over 6 years of age (10–12, 14). Some caregiver heterosurveys assess the child’s symptomatology, particularly those for younger children (13, 15). They are therefore difficult to use at the beginning of placement, as foster families do not know the child. Moreover, we cannot assess which symptoms are reactive or not. Other questionnaires refer to only one traumatic event (10).

Beyond this pitfall in diagnostic terms, the impact of this “not knowing” in the clinic is disconcerting for the therapist and can be a source of misunderstandings. The risks of a story without witnesses have been described in the context of international adoption. The “holes” in children’s stories act like real scotomas, secrets authentically shared by all: the child, his foster family, and the therapist who has the child in charge. Their consequences are less harmful than family secrets, in which there is a conscious desire to hide life events from the child (16). Nevertheless, according to Christian Lachal, in the context of international adoption, “the clinical expression of traumatic events “scotomized” by the rupture of witnesses to a story probably exposes them to a high risk of psychological impasse” (16). Mediatized meetings with parents are therefore essential for the child to reclaim his/her story. The therapist must help the child make connections between elements of incomplete narratives so that he can build his narrative identity (17). The existence of this story, and not its content, therefore plays a fundamental role in child development (18, 19). The narrative of a life allows us to mediate, to weave links between the discordances (ruptures, reversals, and twists) and concordances (overall arrangement) of a life (20, 21). But these scotomas add an element of mystery to this patient care, which exposes us to the risk of filling the gaps with negative representations, of projecting personal fantasies. The lack or absence of an anamnesis is a situation that the therapist may face in other cases, such as with foster children. But the fact that these children were born or taken to an area seen as “enemy,” born of people who may have committed criminal or illegal acts, may lead to a possible fascination or increased fear on the part of the adults around them. These representations can be amplified by the post-attack context and the media. The shock and astonishment following the various attacks in France may reflect a form of suspicion, fear, mistrust, excessive knowledge, or, on the contrary, feeling of incompetence towards these children. In the interest of the child, any professional in charge of these situations must nuance his own representations of parents, i.e., make several narratives co-exist. For example, even if the parents have participated in terrorist acts and are criminals, it seems necessary to be able to assume that some children who have satisfactory psychomotor development have been well carried as babies, in the sense of the holding (22).

We have already listed the possible traumatic events experienced by these children. Upon their return to France, they encounter other difficulties. The filiations of children born in the area, who do not have the documents legally recognized by the French State, are sometimes questioned. Most of the children we met have very full schedules, with multiple appointments (parents in detention, extended families, social workers, etc.) that they may not fully understand. This can lead to a certain confusion linked to the setting and purpose of each appointment and exposes the oldest children to multiple repetitive questions from professionals, on a past that may be painful because it is full of ruptures. During the initial meetings, the child may be suspicious. We spend a lot of time explaining to the children who the different professionals are, where they fit in, and how our role is distinct from the justice system. Indeed, some children may show a certain ambivalence towards professionals in the justice system, as they not only take care of them but also represent a system that deprives them of their parents. In this context, it is even more difficult to establish a diagnosis at the beginning of the assessment. The concept of cultural safety, widely used in Anglo-Saxon countries (23, 24), leads us to question our care practices and the whole system that welcomes the child. The families of these children are French, and have, for the most part, raised these children in a context of migration, rupture of family ties, and in a radicalized ideological and religious context. Often, on their return, the children question the host families, but more broadly the adults around them, about these cultural, religious, and ideological differences. They will be led to crossbreed their logic of representations. We work to accompany them in this process and to help the host families to welcome the child fully, with the contradictions he or she may bring to light.

Upon their return to France, the risk of rupture persists, with some children being moved between several foster families, as have so many other placed children. Another major risk upon their return to France is that of stigmatization. The representations conveyed by some media of these children, seen as “our enemies,” fuel certain societal fears of a supposed risk of future criminal acts. Therefore, the lack of information on their living conditions in the area acts as a real fantastical amplifier, as we have seen. To what extent does the fantasy of dangerousness prevent communication between partners? The different vocabularies used by partners in different professions further increase the risk of misunderstanding. For example, the term “traumatic games” may be differently understood by a judge, a child psychiatrist, or an educator. Additionally, the child’s prognosis, in the eyes of each professional, differs according to his symptomatic reading grid. Another difficulty encountered by the institutions is the question of the transmission of information. What is part of the shared secret? What is part of the medical secrecy? As we have said, we are in contact with host families, educators responsible for child protection, and justice professionals. Every time we meet, on a case-by-case basis, as always in situations involving foster children, we are led to make choices about the relevant information to be communicated in the best interests of the child and which information is more likely to fall within the confidential scope of the interviews. The specificity of this care is the difference in temporality: the timescale of the first assessment of justice is very short compared to the timescale of care. This can lead to more pressing demands from other professionals.

“Trauma is made to be transmitted, this seems to be one of its major characteristics” (25). The effects of trauma on caregivers have been widely studied in the literature, particularly through the lens of counter-transference (26–29). However, the effects of the trauma affect all professionals working with the child, and in the first place, foster families, who are at the forefront of supporting these children. During our encounters with children, we have rapidly become aware of the necessity to accompany fosters families, to help them develop their representations and make sense of the scattered stories of the children. A supporting group was set up for this purpose at the hospital. Training families and professionals on the impact of trauma on childhood development is an essential task of the clinical team. Supervision and analysis of practices are essential to ensure that everyone does not fall into the pitfalls involved (fascination, fear, feelings of incompetence, etc.)

Given the over-publicization of these patients (most of their names have been published in the press) and the number of children concerned, it seems very difficult to publish clinical cases. Patients would be quickly identifiable. Furthermore, epidemiological studies, with standardized scales, are needed in the future to increase the reproducibility of this work. The development of diagnostic tools more adapted to these children to allow a better assessment of their clinical profiles is required. Finally, qualitative research seems indispensable to better identify the representations or counter-transferential issues of the adults surrounding the children. Research in the social and human sciences is needed to refine our knowledge of how children are raised in the area and the specificities of those families who have chosen jihad. Publications exist on individual destinies (30) but do not yet focus on families and how they function.

## Conclusion

The purpose of follow-up, beyond diagnosing and treating disorders, is to attempt to restore meaning and connection in the beginning of a fragmented life. For the narrative to be structuring, it must integrate ambivalence and nuance. Networking with partners (social services, justice, etc.) gradually allows us to try to build a coherent narrative in order to reinforce the narrative identity (17) of these children. The scotomas of their lives, their past in the war area, must not be the breeding ground for stigmatization. Obviously, these children are not responsible for their past; their future is open.

## Data Availability Statement

All datasets generated for this study are included in the article/supplementary material.

## Author Contributions

AK, AlM, MV-M, MF, MC, and FG collected the data. AK and TB designed and directed the project. AK wrote the manuscript with support from AlM, MV-M, FG, JL-B, MF, MC, AnM, JP, SP, and HL-P and under the supervision of DR, TB, MS, and J-MB. All authors discussed the results and approved the final manuscript.

## Conflict of Interest

The authors declare that the research was conducted in the absence of any commercial or financial relationships that could be construed as a potential conflict of interest.
